# CT83 Promotes Cancer Progression by Upregulation of PDL1 in Adenocarcinoma of the Cervix

**DOI:** 10.3390/ijms26062687

**Published:** 2025-03-17

**Authors:** Gilhyang Kim, Kyung-Jun Lee, Eun Shin, Sung Taek Park, Hyeong Su Kim, Hye-Yon Cho

**Affiliations:** 1 Department of Pathology, Kangnam Sacred-Heart Hospital, Hallym University Medical Center, Hallym University College of Medicine, Seoul 07441, Republic of Korea; 2 Department of Pathology, Kangbuk Samsung Hospital, Sungkyunkwan University School of Medicine, Seoul 03181, Republic of Korea; 3 Institute of New Frontier Research Team, Hallym University, Chuncheon 24252, Republic of Korea; rudwns0222@naver.com (K.-J.L.); parkst96@naver.com (S.T.P.); nep2n74@gmail.com (H.S.K.); 4 Department of Pathology, Dongtan Sacred-Heart Hospital, Hallym University Medical Center, Hallym University College of Medicine, Kyeonggido 18450, Republic of Korea; sea4197@hallym.or.kr; 5 Department of Obstetrics and Gynecology, Kangnam Sacred-Heart Hospital, Hallym University Medical Center, Hallym University College of Medicine, Seoul 07441, Republic of Korea; 6 Department of Internal Medicine, Kangnam Sacred-Heart Hospital, Hallym University College of Medicine, Seoul 07441, Republic of Korea; 7 Department of Obstetrics and Gynecology, Dongtan Sacred-Heart Hospital, Hallym University Medical Center, Hallym University College of Medicine, Kyeonggido 18450, Republic of Korea

**Keywords:** CT83, adenocarcinoma, cervix cancer, PDL1

## Abstract

CT83, a cancer-testis antigen, has emerged as a potential biomarker and therapeutic target in various cancers. This study explores its expression and role in cervical adenocarcinoma progression and prognosis. CT83 expression was analyzed in cervical cancer cell lines using quantitative PCR and Western blotting. Functional assays demonstrated that CT83 overexpression (OE) promotes proliferation, migration, invasion, and epithelial–mesenchymal transition (EMT) in cervical cancer cells while also upregulating PD-L1 expression. Conversely, CT83 knockdown reduced these malignant phenotypes. The immunohistochemical analysis of 60 patient samples revealed CT83 expression in 84.9% of cases, with significant correlations to larger tumor size, elevated squamous cell carcinoma antigen (SCC) levels, and advanced FIGO stages (II–IV). Furthermore, intermediate-to-high CT83 expression (H-score ≥100) was associated with more aggressive disease features. These findings suggest that CT83 contributes to tumor progression and immune evasion, likely through PD-L1 modulation. As a highly expressed antigen in cervical adenocarcinoma, CT83 offers promise as a diagnostic marker and therapeutic target for improving patient outcomes.

## 1. Introduction

Cervical cancer is one of the most common gynecologic cancers, with an estimated 604,000 new cases and 342,000 deaths reported in 2020 [1,2]. In the United States, 13,790 new cases of cervical cancer are expected to be diagnosed, and 4310 women were expected to die from the disease in 2023 [2]. The most common histologic type of cervical cancer is squamous cell carcinoma, which accounts for over 70% of all cervical cancer cases and is known to correlate with HPV infection in more than 90% of cases. Adenocarcinoma of the cervix now represents 25% of all cervical cancer cases and is increasingly prevalent among younger women [3]. Other types include adenosquamous carcinoma, small cell carcinoma, clear cell carcinoma, and sarcoma. Notably, approximately 15% of adenocarcinomas exhibit no HPV infection, and women diagnosed with adenocarcinoma tend to have a poor prognosis, even in the early stages of the disease [3]. Existing tumor markers, such as squamous cell carcinoma antigen (SCC) and carcinoembryonic antigen (CEA), are not appropriate for adenocarcinoma [4]. Therefore, developing new tumor markers for the early detection of adenocarcinoma is required.

Cancer-testis antigens (CTAs) are characterized by spontaneous immunogenicity and a unique expression pattern [5]. Generally, CTAs are known to be expressed only in human testis and placental germ cells. Interestingly, some CTAs are highly expressed in tumor cells, suggesting that CTAs might be new biomarkers for prognosis prediction and novel therapeutic targets for treating incurable cancers [6,7].

CT83, also known as KK-LC-1, is one such CTA reported to be highly expressed in various cancers, such as gastric cancer, triple-negative breast cancer, lung adenocarcinoma, and cervix cancer [8,9,10,11]. Recently, T-cell receptor (TCR) gene-engineered T cells targeting CT83 have been developed as a new treatment strategy for various cancers [12].

A study on the immunotherapy of virally induced epithelial cancer revealed that CT83 is expressed in metastatic cervical cancer tissue but not in normal tissue. Moreover, the infusion of CT83-specific T-cell receptor (TCR) gene-engineered T cells (TIL) resulted in the complete regression of metastatic cervical cancer [13]. A phase I clinical trial is underway for T-cell receptor gene therapy targeting KK-LC-1 in gastric, breast, cervical, lung, and other KK-LC-1-positive epithelial cancers [14].

PD-L1 is expressed in 20–80% of cervical cancer cells, contributing to immune evasion by inhibiting T-cell activity [15,16]. Based on the results of the KEYNOTE-158 trial, the FDA approved pembrolizumab, a PD-L1 inhibitor, for treating cervical cancer [17]. Although no direct correlation between CT83 and PD-L1 in immune evasion has been reported, it is evident that both cancer-testis antigens CTAs and PD-L1 play significant roles in enabling tumors to evade the immune system. Their co-expression may foster an immunosuppressive microenvironment that facilitates tumor progression. Targeting both CTAs and PD-L1 could potentially enhance anti-tumor immune responses. For instance, combining CTA-based vaccines with PD-1/PD-L1 inhibitors has reduced tumor burden in solid cancer [11].

In this study, we aimed to evaluate CT83 expression and its role in tumor progression and prognosis in cervical adenocarcinoma. Additionally, we assessed the correlation between clinical factors and CT83 expression in women with cervical adenocarcinoma.

## 2. Results

### 2.1. Expression Pattern of CT83 in Cervix Cancer Cell Lines

**Figure 1:**
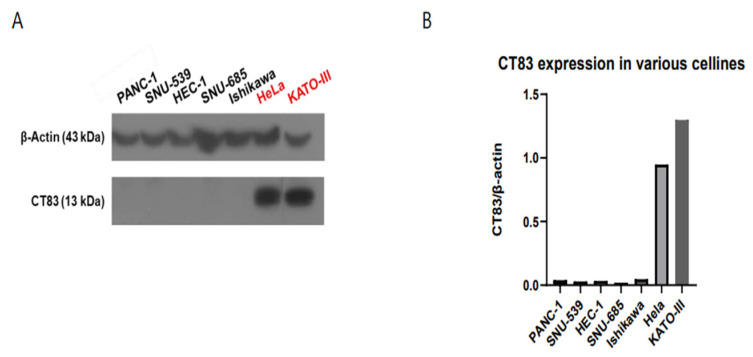
Western blot analysis revealed that CT83 was highly expressed in HeLa cells (cervical adenocarcinoma) and KATO cells (control) (**A**). Quantification of CT83 expression, represented as CT83/β-actin ratio, confirmed relatively high expression in HeLa cells and KATO cells (**B**).

Western blot revealed that CT83 was highly expressed in the HeLa (adenocarcinoma of the cervix) and KATO (control) cell lines (Figure 1A,B).

### 2.2. CT83 Positively Correlates with Cervical Cancer Progression and Invasion

**Figure 2:**
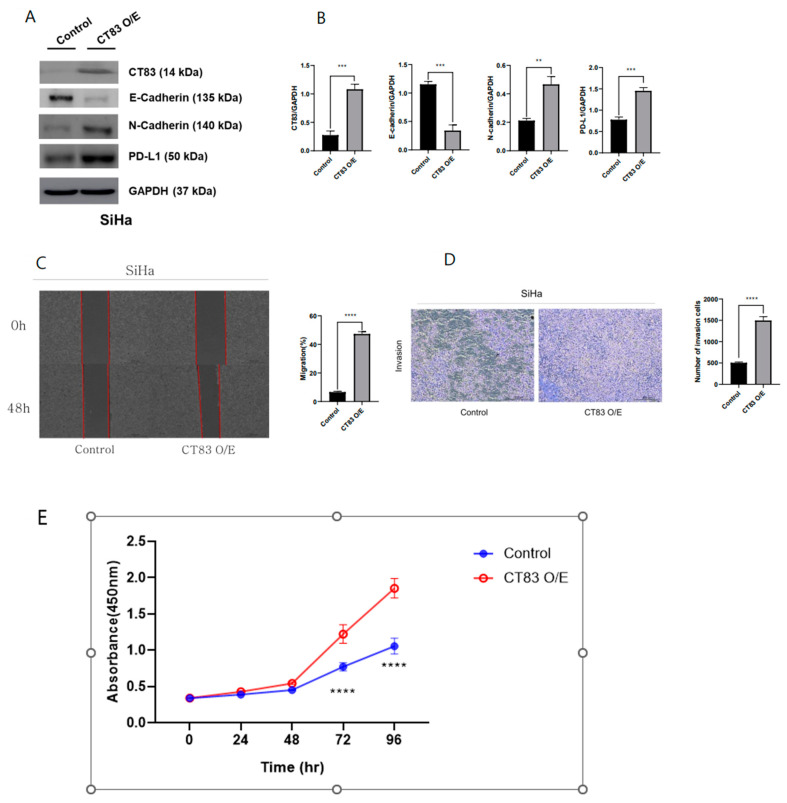
CT83 overexpression (OE) led to increased epithelial–mesenchymal transition (EMT) and PD-L1 expression (**A**). Quantification of CT83, EMT markers, and PD-L1 expression confirmed that the expression of E-cadherin decreased and that CT83, N-cadherin and PD-L1 expression increased in CT83(OE) cells compared with the control (**B**). Additionally, CT83 OE in SiHa cells resulted in enhanced cell migration and invasion rates (**C**–**E**). Significance is indicated as follows: ** *p* ≤ 0.01, *** *p* ≤ 0.001, **** *p* ≤ 0.0001.

**Figure 3:**
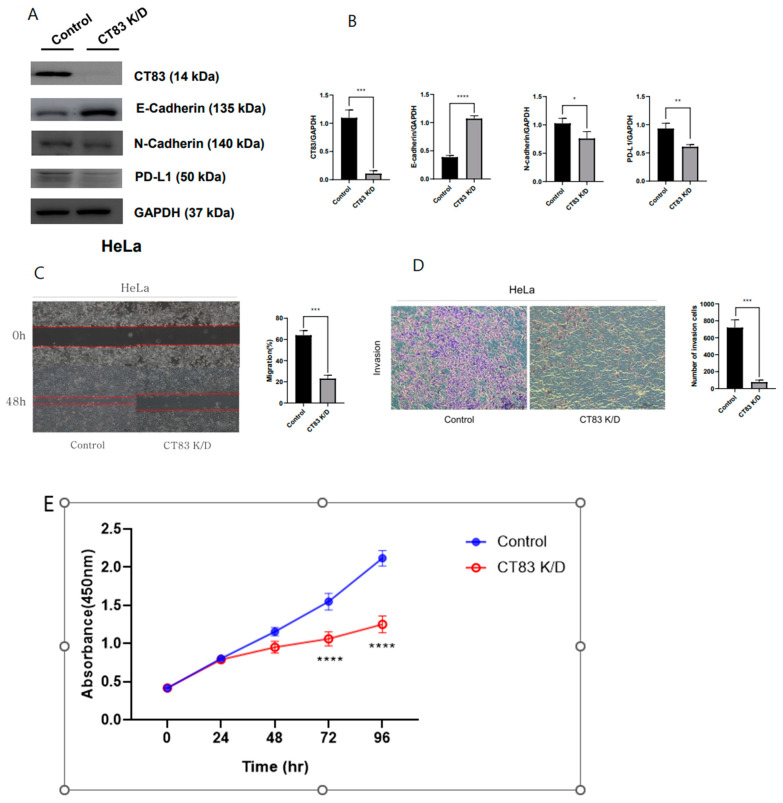
CT83 knockdown (KD) resulted in decreased EMT and PD-L1 expression (**A**). Quantification of CT83, EMT markers, and PD-L1 expression confirmed that the expression of E-cadherin increased while CT83, N-cadherin, and PD-L1 expression decreased in CT83 (KD) cells compared with the control (**B**). Also, CT83 KD reduced cell migration and invasion rates (**C**–**E**). Significance is indicated as follows: * *p* ≤ 0.05, ** *p* ≤ 0.01, *** *p* ≤ 0.001, **** *p* ≤ 0.0001.

To investigate the function of CT83 in cervical cancer, overexpression (OE) and knockdown (KD) studies were conducted in SiHa and HeLa cells, respectively, using Western blot analysis. In the SiHa cells, CT83 OE led to increased epithelial–mesenchymal transition (EMT) and PD-L1 expression (Figure 2A,B). Additionally, CT83 OE in the SiHa cells resulted in enhanced cell migration and invasion rates (Figure 2C–E). Conversely, CT83 KD in the HeLa cells resulted in decreased EMT and PD-L1 expression (Figure 3A,B). Similarly, CT83 KD in the HeLa cells reduced cell migration and invasion rates (Figure 3C–E). Our results suggest that CT83 promotes cancer metastasis, invasion, and potentially contributes to increased apoptosis by facilitating EMT. Moreover, CT83 is associated with PD-L1 expression.

### 2.3. CT83 Was Highly Expressed in Cervical Adenocarcinoma Tissue via IHC Staining

The results of the Immunohistochemistry (IHC) tests showed that 84.9% of samples tested positive for CT83, while 56.6% tested positive for PD-L1. When analyzing the data, we found a weak correlation between CT83 and PD-L1 positivity, with a Pearson correlation coefficient of 0.025. Patients who tested positive for CT83 were significantly more likely to have larger tumor sizes (*p* = 0.046) and higher serum SCC values (*p* = 0.037) (Table 1). Other than these correlations, we did not observe any differences in patient characteristics based on CT83 positivity (Table 1). Additionally, the intermediate-to-high expression of CT83 (H-score ≥ 100) was significantly correlated with the advanced stage of the disease (FIGO stage II~IV) (*p* = 0.046) (Table 2). We also found that a high H-score was correlated with a higher level of serum SCC, but this correlation was not statistically significant (Table 2). Unfortunately, due to a high loss rate to follow-up, we were unable to evaluate survival data.

## 3. Discussion

### 3.1. Summary of Main Results

CT83 is highly expressed in cervical adenocarcinoma cell lines and tissues, with lower expression in cervical squamous carcinoma cell lines.

CT83 overexpression promotes proliferation, migration, invasion, epithelial–mesenchymal transition (EMT), and PD-L1 upregulation. The knockdown of CT83 reduces cancer cell aggressiveness and PD-L1 expression.

Immunohistochemistry showed CT83 positivity in 84.9% of cervical adenocarcinoma samples, correlating with a larger tumor size (*p* = 0.046), and elevated SCC levels (*p* = 0.037). Also, the intermediate-to-high H-score (≥100) was significantly correlated with advanced FIGO stages (*p* = 0.046).

CT83 plays a role in immune evasion by modulating PD-L1 and could serve as a biomarker and therapeutic target for cervical adenocarcinoma.

### 3.2. Results in the Context of Published Literature

Generally, cancer-testis antigens are known to affect cancer growth, proliferation, and metastasis via EMT [18,19]. Also, a study of human liver cancer cell lines showed that CT83 affects EMT markers, such as E-cadherin, N-cadherin, vimentin, and Snail at protein levels [20]. Similarly, in our study, CT83 overexpression correlated with EMT markers’ elevation, while CT83 KD was associated with a decrease in EMT markers. Also, CT83 OE promotes cell migration and invasion, while CT83 KD decreases cell migration and invasion. Based on our results, CT83 may affect tumor growth, proliferation, and invasion via EMT.

We also evaluated the correlation of CT83 and PDL1 (Programmed Death-Ligand 1) in an in vitro study. The correlation between PD1/PDL1 and cervix cancer is well known. PD1/PD-L1 is often overexpressed in cervical cancer, particularly in advanced or metastatic stages, which helps the cancer cells evade immune surveillance by inhibiting anti-tumor T-cell activity. Recently, an anti-PD1 inhibitor drug, pembrolizumab, showed a significant antitumor effect in various cancers, including advanced cervix cancer. The KEYNOTE 158 trial demonstrated a significant antitumor effect of pembrolizumab in treating advanced solid tumors, such as endometrial cancer and cervical cancer [21,22]. Also, the KEYNOTE 82 trial demonstrated the efficacy of pembrolizumab, combined with chemotherapy and optional bevacizumab, in significantly improving the survival outcomes for patients with persistent, recurrent, or metastatic cervical cancer [17].

Interestingly, our data revealed a PD-L1 positivity rate of 56.6% in patients’ tumor tissues, which is higher than previously reported rates. Prior studies have reported PD-L1 positivity in cervical adenocarcinoma ranging from approximately 14.5% to 68% [23,24,25].

In contrast, the other two cited studies included smaller cohorts of adenocarcinoma cases, recurrent disease, and differences in stage distribution, all of which could have influenced the reported PD-L1 positivity rates. These methodological and demographic differences likely account for the observed variability across studies. We believe this variability can be attributed to several factors, including differences in detection methods, quality of tissue samples, sample sizes, interpretation criteria, and racial differences in study populations. For example, a study analyzing 89 tumor tissues from patients with endocervical adenocarcinoma reported a PD-L1 positivity rate of 68%, which is closely aligned with our findings [25]. Notably, this study also utilized the Combined Positive Score (CPS) for PD-L1 assessment, as we did [25].

The relationship between PD-L1 (Programmed Death-Ligand 1) and CT83 in cancer is an emerging area of interest, primarily due to the roles these proteins play in immune evasion and tumor progression. While no direct correlation between CT83 and PD-L1 in cancer has been reported, some studies have observed correlations between other cancer-testis antigens (CTAs) and PD-L1. One study examined the effects of PRAME, a cancer-testis antigen, overexpression in a breast cancer cell line. The authors revealed that a significantly higher proportion of PRAME-overexpressing cancer cells expressed PD-L1. This upregulation of PD-L1 may partially explain the observed reduction in the T-cell-mediated activation and killing of PRAME-overexpressing cancer cells [26]. Interestingly, silencing PRAME resulted in cancer-cell-killing levels like those achieved with anti-PD-L1 atezolizumab treatment [26]. Furthermore, PRAME silencing reduced the frequency of CD8+ T cells expressing the immune checkpoints PD-1, LAG-3, and VISTA, indicating that PRAME tumor expression significantly impairs the PD-L1/PD-1 axis by dysregulating both the ligand and its receptor [26].

In our study, CT83 OE led to increased PD-L1 expression, while CT83 KD resulted in decreased PD-L1 expression. Due to limited prior research, the exact pathway by which CT83 influences PD-L1 expression in cervical cancer cell lines remains unclear. However, several potential pathways have been identified in other cancers, including the epidermal growth factor receptor (EGFR)/ERK signaling pathway, the Janus kinase (JAK)/STAT signaling pathway, and the ERK/mitogen-activated protein kinase (MAPK) pathway [27]. In a clinical setting, no significant correlation was observed between CT83 expression and PD-L1 expression. This discrepancy between the in vitro and retrospective studies may be attributed to differences in tumor samples (e.g., cellular context, sample quality, and racial variations) as well as the tumor microenvironment. Additionally, the limited sample size of the retrospective study may have contributed to the lack of significant correlation. Therefore, further evaluation in a clinical setting is required.

### 3.3. Strengths and Weaknesses

To the best of our knowledge, this is the first study to primarily focus on CT83 expression and its role in cervical cancer cell lines and tissues. We investigated CT83 expression and its role in tumor progression and immune evasion using a robust experimental design that included both in vitro experiments and clinical tissue analysis. Moreover, the link between CT83 and PD-L1 highlights a potential role in immune evasion, supporting further investigation into immunotherapy strategies.

However, a limitation of our study is the lack of mechanistic insights into how CT83 regulates PD-L1 expression and its exact role in immune evasion. Additionally, the small sample size, absence of a control group, and lack of survival data in the clinical tissue analysis are weaknesses. Due to these limitations, we cannot generalize our results to all cervical cancers. Nevertheless, our study underscores CT83 as a potential biomarker in cervical adenocarcinoma, providing a new focus for research and therapeutic targeting.

### 3.4. Implications for Practice and Future Research

The findings from our study emphasize the need for the further exploration of CT83 as a therapeutic target in cervical cancer. Understanding the role of CT83 in regulating PD-L1 expression could enhance strategies for using immune checkpoint inhibitors in treatment protocols. Future research should focus on delineating the pathways involved in CT83′s effects and integrating these findings into clinical trials, paving the way for innovations in cervical cancer management and improving patient outcomes.

## 4. Materials and Methods

### 4.1. Patient Samples

We acquired pathological slides from 60 patients diagnosed with adenocarcinoma of the cervix at Hallym University Medical Center between 2013 and 2024 through a retrospective review of medical records. None of the patients received chemotherapy, radiotherapy, or targeted therapy prior to surgery. Seven of the sixty patients had limited clinical information due to follow-up loss. As a result, while we successfully created tissue microarray (TMA) blocks for all 60 patients, the correlation analysis between the immunohistochemical expression of CT83, PDL1, and clinicopathologic characteristics was only conducted for 53 patients.

This study was performed with the informed consent of all patients and received approval from the Ethics Committee of the Hallym University Medical Center (IRB no. 2023-11-007).

### 4.2. Immunohistochemistry Analysis

To assess the immunohistochemical expression of CT83 and PDL1, we created tissue microarray (TMA) blocks. Tissue cores, 2 mm in diameter, were obtained from donor primary cervical cancer blocks and arranged into recipient TMA blocks using a trephine apparatus (Superbiochips Laboratories, Seoul, Republic of Korea). From each sample, two cores were extracted. Normal cervical tissue was included in the TMA blocks as a control. The TMA slides underwent paraffin removal and rehydration. Endogenous peroxidase activity was quenched using 0.03% hydrogen peroxide, followed by a casein-based protein block (DakoCytomation, Carpinteria, CA, USA) to prevent nonspecific staining. The sections were incubated with antibodies against CT83, PDL1, and thioredoxin (Santa Cruz Biotechnology, Santa Cruz, CA, USA). Sections without primary antibodies served as negative controls. Next, slides were incubated with a horseradish-peroxidase-conjugated secondary antibody (DakoCytomation, Carpinteria, CA, USA) for 30 min, followed by staining with 3,3′-diaminobenzidine for 10 min to produce localized, visible staining. The slides were lightly counterstained with Mayer’s hematoxylin, dehydrated, and mounted with coverslips.

PD-L1 protein expression in cervical cancer was determined by using the Combined Positive Score (CPS), which is the number of PD-L1-staining cells (tumor cells, lymphocytes, macrophages) divided by the total number of viable tumor cells, multiplied by 100. A specimen should be considered to have PD-L1 expression if CPS ≥ 1.

### 4.3. Evaluation of Immunohistochemical Staining

Immunohistochemical expression was quantified by 2 pathologists experienced in evaluating immunohistochemistry. This analysis was conducted in a blind manner. Using H-scoring, we assessed the percentage of stained cells (0% to 100%) and the staining intensity (ranging from category 0 to 3).

The H-score scale ranges from 0 to 300, with specific CT83 expression categories assigned as follows: CT83 low is classified as an H-score of 0 to <100, CT83 intermediate is an H-score of >100 to <200, and high is an H-score of >200 to 300.

### 4.4. Cells and Agents

The Korean Cell Line Bank provided the following cell lines: HeLa and SiHa (cervical cancer), SKOV3 (ovarian cancer), and SNU-685 and SNU-539 (endometrial cancer). The cervical and ovarian cancer cells were cultured in McCoy’s 5A medium (Welgene, Gyeongsan-si, Republic of Korea), while the endometrial cancer cell lines were maintained in RPMI 1640 medium (Welgene). The cell culture media contained 10% fetal bovine serum (FBS, Invitrogen, Waltham, MA, USA) and 1% penicillin (Pfizer, Groton, CT, USA). The cells were incubated in a humidified atmosphere at 37 °C with 5% CO_2_. Transfection reagents (jetPRIME^®^; and Interferin^®^) were obtained from PolyPlus-Transfection (Illkirch-Graffenstaden, France). Antibodies against CT83, E-cadherin, N-cadherin, PDL-1, and actin were sourced from CST (Danvers, MA, USA). CCK-8 assays were provided by Medifab (Geumcheon-gu, Republic of Korea).

### 4.5. Generation of CT83 Overexpression Stable Cell Lines

The Lentiviruses with CT83 overexpression or a control vector were obtained from VectorBuilder, located in Chicago, IL, USA. In 6-well plates, SiHa cell cultures were performed. A medium containing lentiviruses and polybrene (8 µg/mL) was added to the cells at a multiplicity of infection (MOI) of 10 after reaching 60% confluence and thoroughly mixing. After infecting the cells with polybrene to enhance infection, the infection medium was switched to fresh McCoy’s 5A medium after 24 h. Then, 48 h after infection, selection pressure (1 µg/mL puromycin) was applied. Puromycin-resistant cell colonies were selected after 3 to 4 weeks and then cultured in the selection medium to generate SiHa clones from individual cells.

### 4.6. siRNA Preparation and Inhibition of CT83 Expression

CT83 inhibition was induced using a specific siRNA. Both CT83 and control siRNAs were designed and synthesized by Bioneer, located in Daejeon, Republic of Korea. The siRNA duplexes used were as follows: CT83 siRNA, 50-CAG GAU ACU CAG AUG AGUdTdT-30 (sense) and 50-UAC UCA UCU GAG UAU CCU GdTdT-30 (antisense). HeLa cells were placed in 6-well plates at 50% confluence per well and cultured in RPMI-1640 medium supplemented with 10% FBS at 37 °C with 5% CO_2_. siRNAs (50 nM) were transfected using jetPRIME^®^ (Polyplus-transfection, Illkirch-Graffenstaden, France). following the manufacturer’s recommendations. Transfection and silencing efficiencies were assessed using Western blotting.

### 4.7. Western Blot Analysis

Western blot was performed using an SDS–PAGE Electrophoresis System with antibodies specific for KK-LC-1 (Abcam, Cambridge, MA, USA), PD-L1 (Cell Signaling Technology, Beverly, MA, USA), E-cadherin (Cell Signaling Technology, Beverly, MA, USA), N-cadherin (Cell Signaling Technology, Beverly, MA, USA), and GAPDH (Cell Signaling Technology, Beverly, MA, USA). A radio-immunoprecipitation assay (RIPA) buffer from iNtRON, Korea, supplemented with Xpert Protease Inhibitor Cocktail Solution from GenDEPOT, Korea, was used to prepare the total protein lysates. Protein concentrations were determined using a Bicinchoninic Acid assay kit from Thermo Scientific™, Waltham, MA, USA. The proteins were then electrophoretically separated by sodium dodecyl sulfate–polyacrylamide gel electrophoresis and transferred to polyvinylidene difluoride membranes from Bio-Rad, Hercules, CA, USA. The membranes were blocked in 5% skim milk for 1 h and then incubated at 4 °C overnight with primary antibodies against CT83, PDL1, E-cadherin, N-cadherin, and GAPDH. Subsequently, the membranes were incubated with secondary horseradish peroxidase (HRP)-linked secondary antibodies (goat anti-mouse IgG F(ab’)2 pAb (HRP conjugate) (Enzo, Farmingdale, NY, USA, 1:3000) and goat anti-rabbit IgG, pAb (HRP conjugate) (Enzo, Farmingdale, NY, USA, 1:3000)) in 0.5% skim milk at room temperature for 2 h. After incubation, the membranes were washed 3 times (10 min each), and the signals were detected with a Western Pico enhanced chemiluminescent kit (LPS Solution, Burlington, MA, USA), followed by ImageQuant LAS 500 (Cytiva, Marlborough, MA, USA) processing. Image J version 1.51 (National Institutes of Health, Bethesda, Maryland, USA) was used to evaluate band density. CT83, EMT markers, and PD-L1 expression were quantified by measuring their band intensities, normalizing them against a loading control, and comparing between control and experimental groups. We used KATO cell lines (gastric cancer) as the control group for CT83 expression, as CT83 positivity has been reported in up to 79% of early-stage gastric cancer cases [28].

### 4.8. Cell Viability

We assessed cell viability using the WST-8 Viability Assay Kit (Medifab, Seoul, Republic of Korea) by incubating 1500–2000 cells in 96-well plates for 1 day and adding a CCK-8 solution at a 10:1 medium-to-reagent ratio. Absorbance at 450 nm was measured after 2 h. The assays were performed three times to ensure accuracy.

### 4.9. Scratch Wound Healing Assays

Cervical cancer cell migration was examined using wound healing assays. Approximately 5 × 10^5^ transfected or transduced cells were grown in 6-well plates until they reached 100% confluency. The monolayers were wounded by scraping, washed three times with an FBS-free medium, and then cultured in a complete medium. Wound areas were recorded at 0 h and 48 h. Migration was estimated by subtracting the wound areas at 48 h from the wound areas at 0 h. Wound areas were measured using ImageJ software 1.51. Three independent assays were conducted to ensure reliability.

### 4.10. Invasion Assay

The migration and invasion capabilities of cells were evaluated using a transwell chamber with an 8 μm pore size (SPL, Pocheon-si, Republic of Korea) and Matrigel invasion (Corning, Glendale, AZ, USA), respectively. At 48 h post-transfection or transduction, cells in serum-free media were placed in the upper chamber of the transwell, which was coated with 2–10 μg/mL of Matrigel. A medium containing 10% FBS was added to the lower chamber. After 48 h of incubation, the cells that had migrated to or invaded the lower chamber were fixed with 4% paraformaldehyde, stained with 0.1% crystal violet, and counted under a microscope. The number of cells was determined in three independent experiments.

### 4.11. Statistics

The experiments and assays were conducted three times, and data are presented as the mean ± standard error of the mean (SEM). We used Prism (version 8.0) for data analysis. For comparisons between two groups, we used Student’s t-test, and for comparisons among multiple groups, we used a one-way analysis of variance (ANOVA). All tests were two-sided, and significance was set at *p* < 0.05. In all graphs and figures, error bars represent the mean ± SEM from at least three independent experiments or assays. Significance is indicated as follows: NS = non-significant, * *p* ≤ 0.05, ** *p* ≤ 0.01, *** *p* ≤ 0.001, **** *p* ≤ 0.0001.

In accordance with the journal’s guidelines, we will provide our data for independent analysis by the Editorial Team for the purposes of additional data analysis or for the reproducibility of this study in other centers if such is requested.

## 5. Conclusions

Our study provides compelling evidence that CT83 expression is significantly elevated in cervical cancer cell lines and tissues. Notably, CT83 demonstrated the highest expression levels in cervical adenocarcinoma, which was corroborated by the IHC staining of patient tissues. Additionally, we discovered that the OE of CT83 enhances cell migration and invasion through EMT. Furthermore, CT83 OE was associated with increased PD-L1 expression, while CT83 KD resulted in decreased PD-L1 levels, potentially impacting T cell immunity within the tumor microenvironment.

While these findings highlight the significance of CT83 in cervical adenocarcinoma progression, further studies are needed to determine whether similar patterns are observed in other cervical cancer subtypes. CT83 may serve as a potential biomarker and therapeutic target, specifically in cervical adenocarcinoma.

## Figures and Tables

**Table 1:** CT83 expression and clinical characteristics in adenocarcinoma of the cervix.

	CT83 Negative (*N* = 8)	CT83 Positive (*N* = 45)	*p* Value
Age	44.6 ± 9.91	45.0 ± 10.48	0.460
Tumor size	0.8 ± 1.75	2.3 ± 2.24	0.046 *
HR-HPV positive (16/18)	5 (5/8)	28 (28/45)	0.692
FIGO stage			0.194
I	7 (87.5)	29 (64.4)	
II-IV	1 (12.5)	16 (35.6)	
PDL1 CPS	13.8 ± 29.38	8.4 ± 17.96	0.323
PDL1			0.213
Negative	5(62.5)	18 (40.0)	
Positive	3(37.5)	27 (60.0)	
Tumor markers			
CA125	26.2 ± 11.04	50.9 ± 105.9	0.110
CEA	3.0 ± 0.84	46.9 ± 115.32	0.068
SCC	0.7 ± 0.34	1.4 ± 2.01	0.037 *

* *p* ≤ 0.05.

**Table 2:** Expression of CT83 (H-score ≥ 100) and clinical characteristics in cervical adenocarcinoma.

	H-Score < 100 (*N* = 35)	H-Score ≥ 100 (*N* = 18)	*p* Value
Age	45.8 ± 8.24	43.2 ± 13.8	0.488
Tumor size	1.9 ± 2.12	2.4 ± 2.45	0.511
HR-HPV positive (16/18)	22 (22/35)	11 (11/18)	0.624
FIGO stage			0.046 *
I	27 (77.1)	9 (50.0)	
II-IV	8 (22.9)	9 (50.0)	
PDL1 CPS	7.7 ± 15.82	11.8 ± 25.49	0.271
PDL1			0.430
Negative	16(45.7)	7 (38.9)	
Positive	19 (54.3)	11 (61.1)	
PDL1 >10	5 (14.3)	3 (16.7)	0.557
Tumor markers			
CA125	44.7 ± 115.99	55.7 ±53.98	0.383
CEA	46.2 ± 130.40	35.7 ± 69.15	0.411
SCC	1.1 ± 0.79	2.2 ± 3.54	0.072

* *p* ≤ 0.05.

## Data Availability

Data is unavailable due to ethical restrictions.
